# Etymologia: Melioidosis 

**DOI:** 10.3201/eid1707.ET1707

**Published:** 2011-07

**Authors:** Nancy Männikkö

**Affiliations:** Author affiliation: Centers for Disease Control and Prevention, Atlanta, GA, USA

**Keywords:** Etymologia, melioidosis, Whitmore’s disease, glanders

## [me′′le-oi-do′sis]

From the Greek *melis*, distemper of asses, *oeidēs*, resemblance, and *osis*, a suffix indicating an abnormal condition or disease. Alfred Whitmore, a British pathologist serving in Burma, and his assistant C. S. Krishnaswami first described melioidosis in 1912. The infection became known as Whitmore’s disease. In 1925, Ambrose T. Stanton and William Fletcher, the researchers who identified *Burkholderia pseudomallei* as the infection’s causative agent, renamed the infection melioidosis because of its clinical resemblance to glanders.

Sources: Dorland’s Illustrated Medical Dictionary. 31st edition. Philadelphia: Saunders, 2007; Stanton AT, Fletcher W. Melioidosis, a disease of rodents communicable to man. Lancet. 1925;205:10–3. 

